# Impact of different stabilization methods on RT-qPCR results using human lung tissue samples

**DOI:** 10.1038/s41598-020-60618-x

**Published:** 2020-02-27

**Authors:** Margalida Esteva-Socias, Fernando Gómez-Romano, José Antonio Carrillo-Ávila, Alicia Loreto Sánchez-Navarro, Cristina Villena

**Affiliations:** 1 Centro de Investigación Biomédica en Red in Respiratory Diseases (CIBERES), Plataforma Biobanco Pulmonar CIBERES, Hospital Universitari Son Espases, Palma, Spain; 2 Grupo de Inflamación, reparación y cáncer en enfermedades respiratorias, Institut d’Investigació Sanitària de les Illes Balears (IdISBa), Hospital Universitari Son Espases, Palma, Spain; 3 Spanish Biobank Network, Instituto de Salud Carlos III, Madrid, Spain; 4 Andalusian Public Health System Biobank, Granada. Instituto de Investigación Biosanitaria ibs. Granada. Complejo Universitario de Granada/Universidad de Granada, Granada, Spain

**Keywords:** Gene expression analysis, Reverse transcription polymerase chain reaction, PCR-based techniques, Eukaryote, Biomarkers

## Abstract

Aiming to increase the reproducibility of biomedical research results, biobanks obtain human tissues of the highest quality and carry out different storage methods adapted to the needs of analytical technique to be performed by the biomedical researchers. However, there is much controversy and little data concerning the real impact of different stabilization methods on tissue quality, integrity and functionality of derived biomolecules. The influence of four stabilization methods [RNA*later* (RNL), snap freezing (SF), snap freezing using Optimal Cutting Tissue compound (SF-OCT) and formalin-fixed paraffin-embedded (FFPE)] on RNA quality and integrity was evaluated in paired samples of lung tissue. RNA integrity was evaluated through PCR-endpoint assays amplifying six fragments of different length of the *HPRT1* gene and RNA Integrity Number (RIN). To evaluate the difference of tissue functionality among the stabilization methods tested, RT-qPCRs were performed focusing on the differential expression of the *HPRT1*, *SNRPD3* and *Jun* genes. RNA from the samples preserved with the RNL or SF-OCT method showed better integrity compared to SF and FFPE, measured by PCR-endpoint and RT-qPCR assays. However, only statistically significant differences were observed between the RNA from FFPE and other stabilization methods when gene expression of *HPRT1*, *SNRPD3* and *Jun* housekeeping genes were determined by RT-qPCR. For the three mentioned genes, Cq and RIN values were highly correlated. The present work describes the fragility of SF samples, being critical the moment just before RNA extraction, although further experiments of tissue RNA are needed. Standardization pre-analytic workflow can lead to improved reproducibility between biomedical research studies. The present study demonstrated clear evidences about the impact of the stabilization method on RNA derived from lung human tissue samples.

## Introduction

Biomarker discovery with potential clinical application has grown exponentially in the last decades, accompanied also by the evident great biotechnological development in biomedicine. Current research is oriented towards a translational research, personalized medicine and the use of targeted therapies. Therefore, the analysis and validation of biomarkers in large and homogeneous representative cohorts of biological samples with associated data is required, often only possible thanks to collaborative actions between institutions^1,2^. In this scenario, biobanks play a central role in current research, making human biological samples available to researchers, guaranteeing their best quality and associating relevant information to them, integrating clinical, pathological and molecular information and, finally, being a commitment of ethical and legal requirements^3,4^. The objective of biobanks, ultimately, is to ensure that samples provided to researchers are fit-to-purpose, which means that they have an adequate quality to perform the analysis for those they have requested biospecimens.

There are many factors that contribute to a biological sample for being considered of suitable quality, among them, those involved during the pre-analytical stage. Despite having identified some of the main pre-analytical contributors to the loss and quality assurance of human biospecimens, there is still not enough clear information available to know to what extent each of these factors contributes. Aiming to clarify this gap in knowledge, a new discipline called Biospecimen Science (BS) emerged in the field of biobanks focused on studying how pre-analytical factors may alter integrity of samples^5^. It also focuses on discovering new quality biomarkers that could help us to assess quality of human biospecimens using standard and validated analytical techniques^6,7^.

In the last years, there has been an increase of scientific interest based on RNA analysis^8^ enabling the development of new techniques used for transcriptome-based studies. Consequently, BS has been immersed on studying the possible effects of pre-analytical variables on RNA derived from human tissue samples. The parameters studied range from the moment in which tissue is obtained during surgery to the moment in which researchers carry out experimental procedures. All pre-analytical parameters included in this range could alter the integrity of biomolecules contained in tissues and thus also introduce a bias in the results obtained. For example, it has been demonstrated that ischemia time (IT), both warm and cold, as one of the most influencing factors on RNA integrity^9–11^. However, no consensus has yet been reached to determine the exact moment in which RNA quality and integrity begin to decrease. Probably this fact is due to the interaction of other pre-analytical factors such as preservation type^12^, preservation method^13^, long term storage^14,15^, RNA extraction methodology^16^, among others.

Most of the factors mentioned above do not depend on human activity, which means that they are practically unavoidable. However, how human tissue samples are stored and stabilized could help to fit them to different research purposes. In order to shed light into this issue, the effect of each stabilization method on the quality of human tissue samples and the RNA derived from it should be studied. In most cases researchers do not have fresh tissue availability and, as a result, most studies published to date have focused on comparing the quality of RNA obtained from frozen and formalin fixed samples. It is known that RNA derived from FFPE samples has undergone cross-links, chemical modifications and degradation over time due to the fixation method^17^. These molecular changes support that better quality is usually determined in RNA derived from frozen in comparison with FFPE samples^18,19^ although in some cases RNA derived from FFPE was reported to be suitable for gene expression experiments^20^. Today, there are alternative choices for long-term storage of human tissue samples, including RNA preservation solutions. However, no clear evidence has been determined regarding quality and integrity of RNA when using alternative stabilization methods.

Because of these worrying antecedents and the need to advance in biomedicine, the present study aimed to identify possible effects or alterations on RNA quality and integrity on gene expression caused by four different widely used stabilization methods, including both formalin fixative and formalin-free preservation methods.

## Results

### RNA integrity differences between stabilization methods

**Figure 1:**
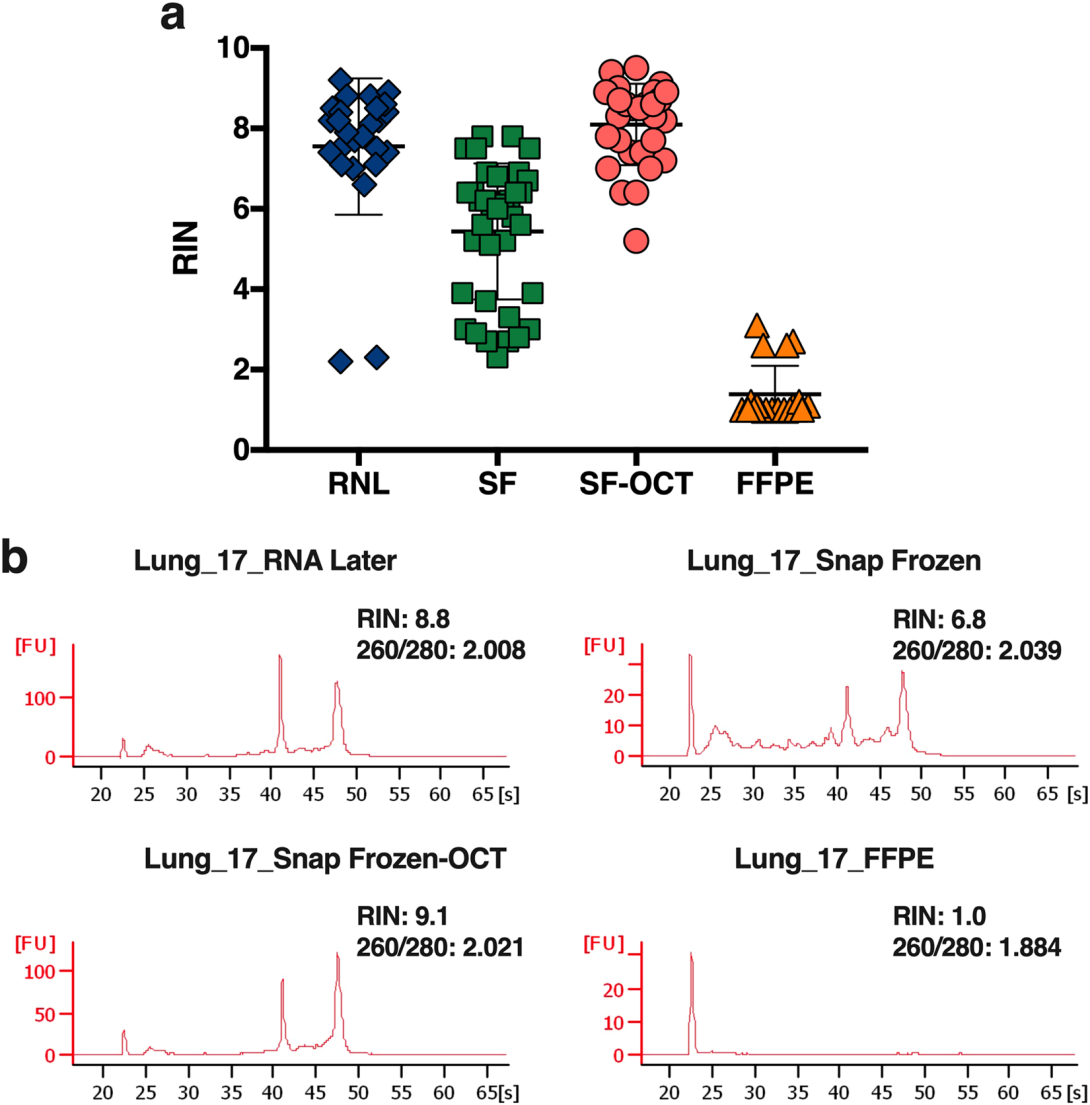
RNA quality assessment by RNA Integrity Number (RIN). (**a**) RIN values obtained for each sample according the stabilization method used. (**b**) Electropherograms of the RNA extracted from lung tissue samples from patient 17.

A total of 104 lung tissue samples from 20 patients were analysed to evaluate the impact of different stabilization methods on RNA integrity. RIN average and standard deviations (SD) were calculated (Table 1) for each stabilization method tested, which are plotted in Fig. 1a. Lower integrity is observed for RNA samples from SF and FFPE, with a RIN average of 5.2 and 1.4, respectively. The highest RIN values were obtained in the case of RNL (7.6) and SF-OCT (8.1) tissues.

**Table 1:** Overview of RIN parameters to assess RNA quality.

Stabilization method	RIN Average	SD^a^
RNA*later*	7.6	1.7
Snap Frozen	5.2	1.7
Snap Frozen-OCT	8.1	1.0
FFPE	1.4	0.7

^a^Standard deviation.

These differences were also detectable if considering each particular patient. Figure 1b shows electropherograms obtained for the RNA extracted from the patient 17, that was randomly selected. There are clearly differences between the RNA from the different conservation protocols: a RIN of 8.80 were obtained for RNL, 6.80 for SF, 9.10 for SF-OCT and 1.0 for FFPE. The RNA purified from RNL and SF-OCT showed sharply defined 18 S and 28 S rRNA peaks. For SF, sample peaks were also distinguishable, but there was an increase of signal in the pre-, fast- and inter- regions due a slight degradation. Nevertheless, rRNA peaks from FFPE lung tissue samples were not detectable in electropherogram analysis. The 260/280 ratios were around 2.0 in all cases, except in the case of FFPE suggesting lower purity of the RNA extracted in comparison with the other procedures.

### Effect of the stabilization procedure on different length fragment amplification capability

**Figure 2:**
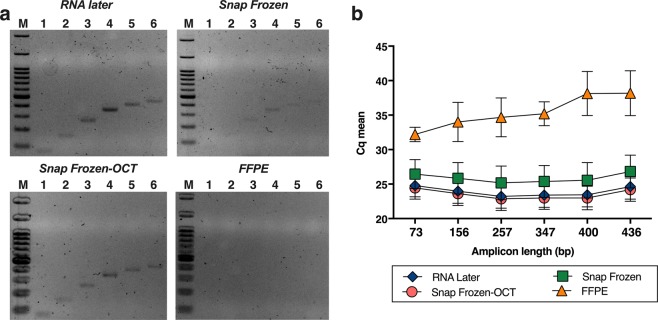
Functional RNA quality assessment by fragment size PCR Endpoint assay and qRT-PCR technique. Six amplicons of different length (73, 156, 257, 347, 400 and 436 bp) of the *HPRT1* gene were amplified using cDNA purified from RNL, SF, SF-OCT and FFPE lung tissue samples (n = 20). (**a**) Images of electrophoresis gels showing the PCR products for the six amplicons tested for patient 17 in all preservation methods compared: RNL (top of the left), SF (top of the right), SF-OCT (bottom of the left) and FFPE (bottom of the right). Full-length gels are presented in Fig. S1. (**b**) Cq mean values and standard deviation (SD) obtained by each amplicon in each preservation methods tested (n = 20). SDs are represented as error bars. For RNL, SF and SF-OCT, SD was too small to be consistently represented in the graph.

In order to evaluate the integrity and RNA fragmentation of lung tissue samples preserved in the mentioned procedures, extracted RNA was amplified for 6 different amplicon lengths from the *HPRT1* gene. PCR Endpoint products were loaded in an agarose gel (Fig. 2a) to observe differences between procedures in terms of presence or absence of electrophoretic bands. When using RNA extracted from RNL and SF-OCT tissues amplification of all fragments was observed consistently. However, in the case of RNA from SF tissue, a very low signal was obtained from all the bands, and none from FFPE samples. At this point, we hypothesized that RNA quality from SF and FFPE were not high enough to visualize bands by an electrophoresis gel due to a low amount of PCR products generated.

Consequently, aiming to respond the previous findings, we performed the assay of differential amplicon length on a CFX96 Real-Time PCR Detection System. This platform allowed us to determine differences between the conservation procedures tested and their possible effect on amplification outcome of the extracted RNA in terms of Cq values.

The results obtained for each of the amplicons using qPCR are presented in Fig. 2b. Similar results were obtained regarding RNA from RNL and SF-OCT tissue, with an average of 24 cycles for both the first and sixth amplicons. But higher values were obtained for the SF samples, leading to results in line with those obtained by PCR Endpoint assay. The Cq mean values were 26 for the 73 bp amplicon and 27 for the 436 bp amplicon. In contrast, for RNA from FFPE showed Cq values between 30 and 33 in the smallest fragment and between 32 and 40 for the longest.

### Analysis of gene expression levels of housekeeping genes as a RNA functional integrity testing of tissue samples

**Figure 3:**
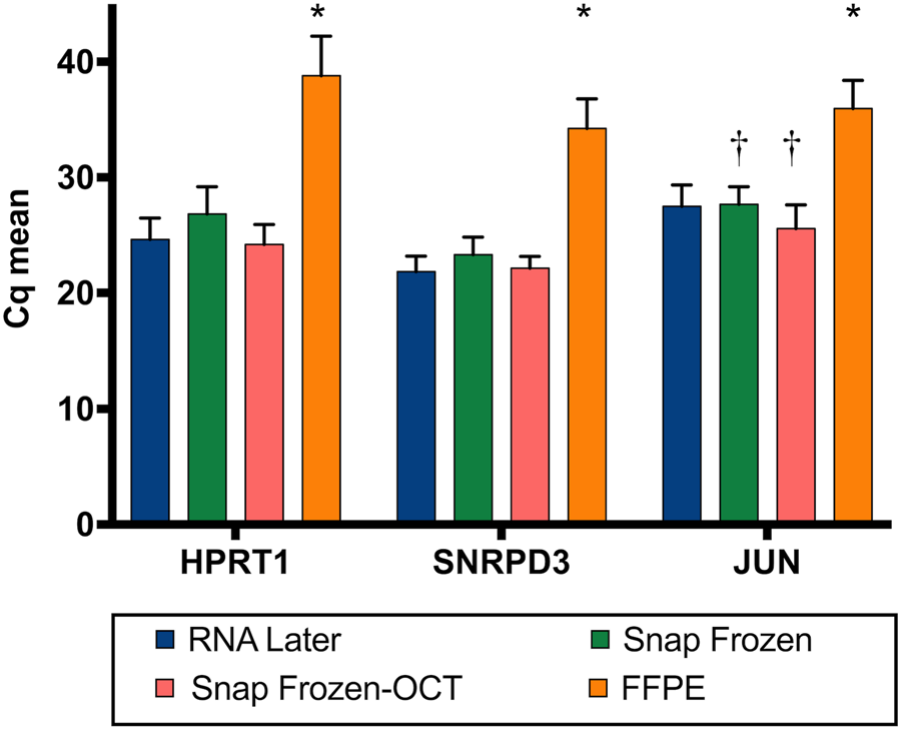
Gene expression levels on lung tissue samples stored with different preservation procedures. RNA isolated from RNL (blue), SF (green) SF-OCT (salmon) and FFPE (orange) was reverse transcribed into cDNA and analysed by qPCR using primers for *HPRT1*, *SNRPD3* and *Jun* genes. * indicates significance at p < 0.0001 and † at p < 0.005.

The use of RNA to perform gene expression analysis through RT-qPCR has become a routine analysis in biomedical research. Therefore, the next objective was to evaluate if the different preservation procedures could have any possible effect on the expression levels of three housekeeping genes. To ensure unbiased 3′ and 5′ coverage we used anchored oligo dT and random hexamer primers for cDNA synthesis. We obtained Cq values from all patients and stabilization methods using primers for *HPRT1*, *SNRPD3* and *Jun* genes and SYBR-green based assay. Cq values obtained for each experimental condition are shown in Fig. 3 and Table 2. The lower Cq values for *HPRT1* gene were obtained with SF-OCT samples, ranging from 21.69 to 27.27. However, for *SNRPD3* gene, RNL samples showed the higher gene expression levels, with Cq values between 19.63 and 24.38. Finally, SF-OCT also showed the lower Cq values for *Jun* gene expression (ranging from 22.83 to 32.36 cycles). Amplification plots for the mentioned reference genes are shown in Fig. S2, where clear difference can be observed between RNA purified from FFPE and SF in comparison to RNL and SF-OCT.

**Table 2:** Summary of the data obtained in functionality RNA analysis by of RT-qPCR.

Stabilization method	HPRT1				SNRPD3				Jun			
	RNL	SF	SF-OCT	FFPE	RNL	SF	SF-OCT	FFPE	RNL	SF	SF-OCT	FFPE
Cq mean	24.64	26.82	24.19	36.47	21.84	23.32	22.13	34.24	27.48	27.66	25.55	35.97
SD	1.84	2.37	1.70	2.44	1.34	1.51	1.03	2.56	1.85	1.53	2.08	2.42
Minimum Cq	21.87	23.51	21.69	32.47	19.63	21.16	20.12	31.52	23.81	24.35	22.83	32.12
Maximum Cq	29.91	32.68	27.27	41	24.38	27.54	24.18	39.38	30.91	31.32	32.36	39.80

RNA levels on FFPE samples were statistically lower than RNL, SF and SF-OCT expression thus presenting significant higher Cq values in comparison to other procedures (p<0.0001). However, no statistically significant differences were found among RNL, SF and SF-OCT samples in the tested genes, except for *Jun* gene expression, being slightly significant lower on SF compared to SF-OCT samples (p<0.005).

RNL and SF-OCT apparently preserve more efficiently quality and integrity. To clarify if there is any effect during the period from thawing until the sample is immersed in Qiazol or a similar reagent, a time course of thawing tissue at room temperature (RT) were designed to evaluate gene expression levels of *HPRT1*, *SNRPD3* and *Jun* genes. The experiment was performed using RNL, SF and SF-OCT samples from 4 organ donors already included in the previous experimental design. Tissue aliquots or sections were submitted to RT during 0, 1, 3, 6, 12, 30 and 45 minutes. Once time had elapsed, samples were immersed in Qiazol and RNA was extracted.

**Figure 4:**
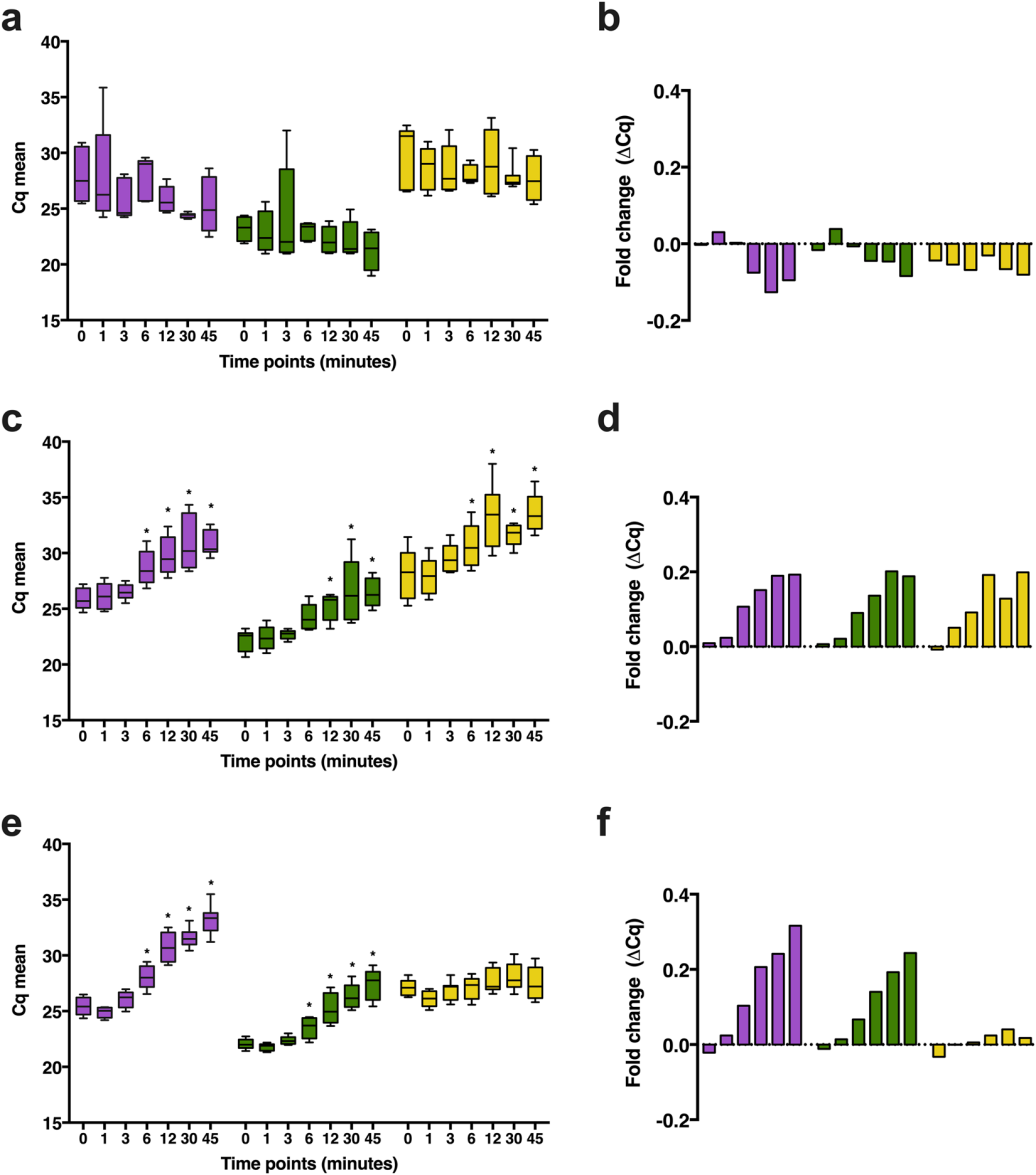
Expression levels of housekeeping genes when evaluating the impact of thawing time before RNA extraction. RNL (**a**,**b**), SF (**c**,**d**) and SF-OCT (**e**,**f**) tissue samples were submitted to increasing thawing times (0, 1, 3, 6, 12, 30 and 45 minutes) at room temperature and Cq values of *HPRT1* (purple), *SNRPD3* (green) and *Jun* (yellow) genes were measured. Time points were compared using two-way ANOVA with multiple comparisons. All time points highlighted with *showed a significant difference compared to the control condition (0 minutes) with a p < 0.01.

RNA from samples preserved in RNL did not present differences in terms of expression levels in any of the three genes studied (Fig. 4a). When fold change was calculated, considering time 0 as control, minimal or negative changes were observed indicating Cq values obtained at the basal time point were not exceeded even after 45 minutes at room temperature (Fig. 4b). Instead, RNA obtained from SF samples showed increasing Cq values the longer the samples were submitted to RT (Fig. 4c,d). When *HPRT1* expression levels were evaluated, a significant increase in Cq values was observed after 6 minutes in comparison to time 0. For *SNRPD3* and *Jun* genes, statistically significant changes begin after 12 minutes. Finally, as shown in Fig. 4e, increasing amplification cycles were obtained when the *HPRT1* and *SNRPD3* genes were analysed, with the changes being statistically significant after 6 minutes. However, these changes were not observed for the *Jun* gene, which showed a minimal change rate with respect to the basal time (Fig. 4f).

RNA integrity was also checked with Bioanalyzer 2100 (Agilent). RNA from RNL preserved tissue showed a stable RIN among time, being consistent with the findings observed by qPCR, described above. Conversely, RIN values obtained from SF and SF-OCT showed a reduction of 0.5 and 0.7 after 45 minutes at RT on RIN value, respectively (Fig. S3).

### Correlation between Cq values and RNA integrity

**Figure 5:**
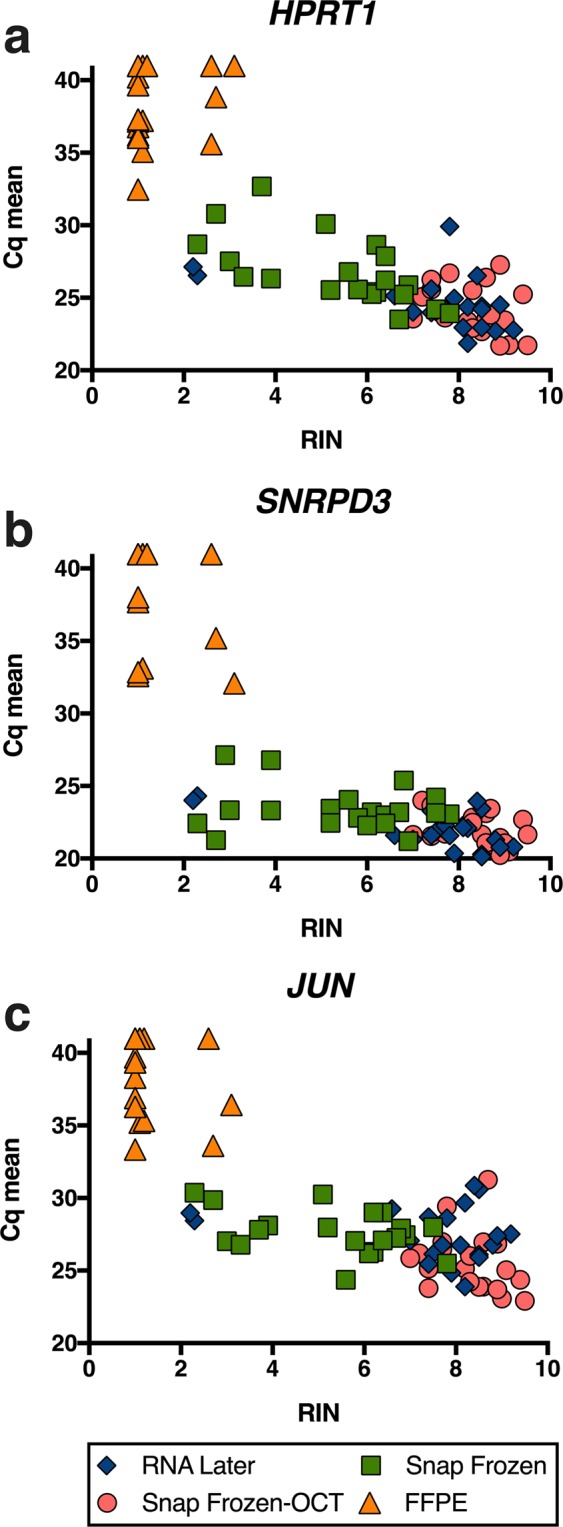
Comparison of RIN values and RT-qPCR results of lung tissue samples according their stabilization method. RIN and Cq values by RT-pPCR of RNA extracted from 80 different samples of 20 patients are shown for (**a**) *HPRT1*, (**b**) *SNRPD3* and (**c**) *Jun* genes.

Results obtained from the RT-qPCR and Bioanalyzer 2100 were plotted on three independent graphs (one per each gene studied) and nonparametric Spearman correlation was performed to better elucidate the probable relationship between the two metrics (Fig. 5). Overall, clusters were observed according the stabilization protocol used in the three genes evaluated. Samples with higher Cq values were those that also have lower integrity according to their RIN value measured from 0 to 10. The correlation measured by R Spearman index between Cq mean and RIN values was of −0.7379 for *HPRT1*gene, −0.8011 for *SNRPD3* gene and −0.7252 for *Jun* gene (p < 0.001).

## Discussion

The development of new disease biomarkers strongly depends on using high quality samples and their associated data. Biobanks are currently the main tool to guarantee this requirement due to their close relation with clinical routine and biomedical research. However, the control and recording of pre-analytical variables is one of the biggest challenges when trying to guarantee sample and data quality. Moreover, this difficulty is further compounded when handling with different sample types, tissues obtained through different surgical procedures (biopsy, resection or *post mortem*) which are going to be tested through a wide variety of analytical techniques.

In that context, the present work aimed to compare integrity, quality and functionality of RNA derived from lung tissue preserved by four of the most widely used stabilization methods in tissue biobanking, Pathology Departments and biomedical research laboratories (RNL, SF, SF-OCT and FFPE). To perform the study, a total of 20 paired lung tissue samples were studied to rule out both intra and interindividual variations. We have evaluated the impact of different long storage methods for preserving lung tissue on integrity and quality of RNA using standard analytical techniques, such as Bioanalyzer 2100 and RT-qPCR. To achieve this, in addition to using samples from conventional surgery (n = 16), samples from organ donations (n = 4) had also been included, since large amounts of tissue can be collected, which was essential to carry out the present study. Nevertheless, after the first results, we made sure that there were no biases in the inclusion of both patient profiles (Fig. S4).

The most common way to measure the integrity of RNA is calculating the RIN, based on the ribosomal RNA signal, that takes into account their peaks, positions and separation times of three different regions (fast, inter and post). Therefore, the final RIN comes from the combination of the relative weight of each of these regions^21^. We have detected clear differences among RIN results when comparing the four stabilization methods, which suggests that RNA behaves differently under these conditions. The use of paired samples for all stabilization methods enables us to ensure that the differences observed are not due to the pre-analytical variables associated with clinical or collection features, but specifically to the stabilization method. Accordingly to Freidin *et al*.^22^, the lowest RIN values have been obtained for FFPE samples in comparison with non-fixatives stabilization methods.

To investigate whether observed differences by RIN means RNA functional changes, we performed an amplicon length assay to test the loss of electrophoretic bands and the increment of Cq values among preservation methods targeting the housekeeping gene *HPRT1*. At this point, we have obtained concordant results with those recorded before, thus showing that lower RIN values translate into loss of amplification capacity measured by the low signal or absent electrophoretic bands and the increase of Cq values in RT-qPCR. For example, Kashofer *et al*.^18^ previously correlated Cq and RIN values obtained from cryopreserved and FFPE samples using thyroid gland, kidney and duodenum tissues.

We decided to use RT-qPCR, since it is a technique widely used to test gene expression in a more precise, simple and cost effective way compared to other alternative techniques, such as arrays, which show a high variability in signal-to-noise ratio, require properly trained staff and it is more expensive^23^. We hypothesized *HPRT1* results obtained by RT-qPCR should be comparable to other housekeeping genes, such as *SNRPD3* or *Jun*. All three genes showed the same expression pattern, being FFPE the samples with significant higher Cq values followed by SF, RNL and SF-OCT, respectively. These results are in agreement with those presented by Wimmer *et al*.^19^, who also observed a clear separation between SF and FFPE samples, both in different Bioanalyzer profiles and the higher Cq values observed with *GAPDH*, *G6PD* and *SDHA* reference genes. Therefore, we present reproducible results even though we tested different reference genes.

An important issue to take into consideration is that formalin forms irreversible cross-links between proteins, which maintain the structural integrity of the cells^24^. However, cross-linking has a deleterious effect on nucleic acids, so extracting intact nucleic acids from these types of samples is a challenge, since they suffer damage and severe degradation in the preparation and the storage process of the sample. Historically, the purification of nucleic acids from FFPE samples has been a difficult procedure, mainly due to the inefficiency of the binding reversal process. This often contributes to the degradation of nucleic acids and directly affects the performance of the following stages^12,25^, which could explain the findings mentioned above. Some variables that could improve the quality of biomolecules derived from FFPE, such as incubation temperature^26^, are under investigation. But there are still no validated alternative procedures, except for alternative fixatives such as Methacarn^27^, PAXgene^28^ or paraformaldehyde^29^, among others.

During the last years, snap freezing has been established as a gold standard for RNA preservation in tissue specimens^30^ and, consequently, for gene expression assays when there is no accessibility to fresh tissue. Despite this, our results suggest that SF is less effective than RNL or SF-OCT preserving RNA although there are not statistically significant differences and further experiments are needed. Similar results were published by Keating *et al*.^31^, when comparing the ability of PBS, Trizol, RNL and SF to preserve nucleic acids for microarray chip analysis. The authors proposed that tissue immersion in a liquid such as RNL, besides preserving RNA, is a mechanism that promotes the elimination of residues and RNases in the lower part of the cryovial, a process that is not carried out when the tissue is snap frozen in liquid nitrogen.

We firstly hypothesized that RNL and OCT compounds could play a protector role during storage time in ultrafreezer at −80 °C, unlike SF samples in which tissue are not embedded in any reagent. This could explain the differences observed between the three stabilization methods. In order to shed light into this issue, we performed a time course experiment of thawing tissue at RT. Expression levels of *HPRT1*, *SNRPD3* and *Jun* genes were evaluated for all RNA samples subjected to increasing thawing RT times up to 45 minutes. This experiment clearly showed the stability of RNA with RNL preservation across time in comparison with SF and SF-OCT, where significant increases of Cq values were detectable after 6 or 12 minutes of incubation at RT. In view of this, we assumed the effects on RNA quality observed may be due to the preservation properties of RNA*later* reagent, since the RNA from SF-OCT tissue blocks did not showed stable quality over time. It seems believable that SF and SF-OCT tissue are degraded immediately, destroying intracellular compartments, making accessible all cellular content to RNases while this protection is stable when RNA*later* is used^32^.

In conclusion, all results presented here lend weight to the argument about the lower quality of the formalin fixed samples compared to those frozen, whether RNL, SF or SF-OCT are used. Although no statistically significant differences have been observed between frozen stabilization methods, a slight better RNA stability and integrity were observed in RNL samples. Despite sample size limitations, our study provides plausible evidences for considering Bioanalyzer 2100 and RT-qPCR results as comparable methods for assessing RNA quality, in accordance with Walker *et al*.^33^. Furthermore, it is important to note the value of selecting reference genes to conduct the experiments, i.e. the selected genes must have a constitutive expression in every cell, to perform gene expression studies using samples with different stabilization methods. The findings in *Jun* gene in the time course experiment using SF-OCT samples support the idea about differential sensitivity and stability of reference genes to RNA degradation and their variation within the same tissue as it was discussed in Pérez-Novo *et al*.^34^. Given the above, we consider that control of pre-analytical factors is essential to ensure the best quality assessment of RNA, especially when samples have been stored by different stabilization methods, thus introducing a clear bias on research results.

## Materials and Methods

### Sample collection, fixation and storage

Lung tissue samples were supplied by the Pulmonary Biobank Consortium from surgical excisions and organ donation carried out at Hospital Universitari Son Espases (HUSE). Non-pathological (and non-tumoral) lung tissue samples were stored and pre-analytical variables registered according to SPREC codification^35^. Lung tissue samples from twenty participants with no history of COPD, asthma or OSA and no bronchodilator or corticoid therapy were included. All the cases presented a normal histology and the cold ischemia time (CIT) was lower than 30 minutes. Clinical, pathological and pre-analytical characteristics of all patients enrolled are provided on Table 3. The study was approved by the Human Research Ethics Committee of the Balearic Islands. All the participants, or familiars in case of organ donors, authorised to participate by written informed consent for both biospecimen and clinical information collection. The study conformed with the Helsinki declaration (1964) and its amendments or comparable ethical standards.

**Table 3:** Outline of clinical and pathological characteristics of recruited patients and their pre-analytical associated data.

Variable	Total (±SD^a^)	Percentage (%)
Age (years)	62.8 ± 14	
**Gender**		
Male	15	75
Female	5	25
**Smoking habit**	13	65
Pack-year >20	2	15.4
Pack-year <20	11	11
**Lung cancer**	15	75
Squamous cell carcinoma	1	6.7
Adenocarcinoma	10	66.7
Carcinoid tumor	3	20
Small cell-carcinoma	1	6.7
**Cancer staging**		
IA-IIIA	11	73.3
IIIB-IVB	4	26.7
**Type of collection**		
Surgical excision	16	80
Warm ischemia time (min)	26.5 ± 26.02	
Cold ischemia time (min)	18.69 ± 5.56	
Organ donation	4	20
Warm ischemia time (min)	119.67 ± 89.28	
Cold ischemia time (min)	14.75 ± 5.32	
**Fixation time of FFPE**^**b**^ **samples (h)**	37.58 ± 14.15	
**Sample age (storage time)**		
<1 year	11	55
1–2 years	5	25
2–5 years	4	20

^a^Standard deviation, ^b^Formalin fixed, paraffin embedded.

In the case of smokers, cumulative smoking exposure was determined in terms of pack-years by multiplying the number of years smoked with the average number of packs per day.

Four different procedures of tissue preservation were compared. Up to nine specimens per condition were preserved in (i) a 1,8 ml cryotube and snap frozen in liquid nitrogen (SF), (ii) 500 µl RNA*later* Stabilization Solution (Invitrogen) (RNL), (iii) Tissue-Tek O.C.T. compound (Sakura) and frozen in an isopentane bath (SF-OCT), and (iv) 4% neutral buffered formalin. RNL samples were incubated 24 hours at 4 °C and then placed at −80 °C. Formalin fixed specimens were under fixation for at least 24 hours at room temperature, dehydrated with a Leica TP1020 tissue processor and then were paraffin embedded. SF and SF-OCT samples were immediately placed in a −80 °C freezer.

### RNA extraction and cDNA synthesis

Two extractions methods were performed depending on the preserved type of samples. On the one hand, 3 sections of 10 µm thick of formalin fixed paraffin embedded (FFPE) tissue were deparaffinated using 320 µl of Deparaffinization Solution (Qiagen, Hilden, Germany). Total RNA was extracted with the RNeasy FFPE Kit (Qiagen, Hilden, Germany) according to the manufacturer’s instructions. And on the other hand, in the case of frozen samples, a range of 12.4–61.7 ng of tissue samples preserved in RNL, a range of 29–54.4 ng of SF tissue or 20 sections of 20 µm of SF-OCT were disrupted by Tissue Lyser LT (Qiagen, Hilden, Germany) using 700 µl of QIAzol as lysis reagent and one stainless steel bead of 500 mm diameter. Total RNA was extracted using miRNeasy mini kit (Qiagen, Hilden, Germany) following the manufacturer’s instructions. The concentration and purity of each RNA sample (Table 4) were assessed using Nanodrop spectrometer (Thermo Scientific, Wilmington, DE) and RNA integrity score were obtained by RNA 6000 Nano assay and 2100 Bioanalyzer Instrument (Agilent Technologies, Wilmington, DE). After characterization, purified RNA samples were stored in 5 µl aliquots at −80 °C until analysis.

**Table 4:** Results of the quality and quantity of RNA samples obtained according its preservation procedure.

	RNA*later*	Snap Frozen	Snap Frozen-OCT	FFPE
Starting material (mg of tissue)	38.31 ± 12.54	46.69 ± 23.00	20 sections of 20 µm	3 sections of 10 µm
Elution volume (µl)	50	50	50	30
Concentration (ng/µl)	400.80 ± 189.28	484.62 ± 210.19	302.81 ± 240.22	34.28 ± 16,03
A260/280	2.009 ± 0.018	1.999 ± 0.068	1.979 ± 0.103	1.858 ± 0.069
A260/230	1.804 ± 0.355	1.842 ± 0.195	1.448 ± 0.460	1.552 ± 0.410

Results are presented as average ± SD.

For analysis, cDNA was transcribed from 100 ng of total RNA, using the SensiFAST cDNA Synthesis Kit (Bioline) following the manufacturer’s protocol. Reactions were performed in a final volume of 20 µl, using 4 µl of buffer and 1 µl of reverse transcriptase provided by the supplier. Reverse transcription reactions were performed in a T100 Thermal Cycler (Bio-Rad) as follows: 10 minutes at 25 °C for primer annealing, followed by 42 °C for 15 minutes in order to reverse transcription and 85 °C for 5 minutes for enzyme inactivation. All cDNA samples obtained were diluted into 5 volumes of doubly distilled water and preserved at −20 °C.

### Fragment size PCR Endpoint assay

We stablished an assay based on the housekeeping gene Hypoxanthine Phosphoribosyltransferase 1 (*HPRT1*) fragment amplification because of its constitutive expression among human tissues according to Human Protein Atlas. We amplified 6 amplicons of different length (73, 156, 257, 347, 400 and 436 bp), using a common forward primer and six reverse primers (Table S1) designed with the Primer-Blast tool. This strategy was previously described by Kashofer *et al*.^18^ using *GAPDH* gene.

Endpoint PCR were performed using the FastStart High Fidelity PCR System (Roche) in a final volume of 50 µl containing: 5 µl of FastStart High Fidelity Reaction Buffer 10×(with 18 mM MgCl_2_), 1 µl of DMSO, 5 µl of each primer (final concentration 0.4 µM), 1 µl of PCR Grade Nucleotide Mix (final concentration 200 µM each dNTP), 0.5 µl of FastStart High Fidelity Enzyme Blend (5 U/µl), 2 µl of diluted cDNA and 30.5 µl of PCR-grade water. The PCR reaction was performed in T100 Thermal Cycler (Bio-Rad) following the next steps: initial denaturation at 95 °C for 2 minutes, 35 amplification cycles (denaturation at 95 °C for 30 seconds, annealing temperature [Table S1] for 30 seconds and an elongation step at 72 °C for 2 minutes) and final elongation step at 72 °C for 5 minutes.

Amplification products were separated into 2% agarose gel (Conda Laboratories) on 1X TAE Buffer (Invitrogen) at 100 V for 30 minutes in a RunOne Electrophoresis Cell (EmbiTec, San Diego, CA). SYBR Safe (Invitrogen) was added to agarose gel according to supplier recommendations to visualise the DNA bands on ImageQuant LAS 4000 (GE Healthcare Life Sciences).

### RT-qPCR

For functional assessment of RNA quality, quantitative Polymerase Chain Reaction (qPCR) experiments were performed for *HPRT1*, *SNRPD3* and *Jun* housekeeping genes. Primers were also designed with the Primer-Blast tool available online (Table S1). We used the common forward primer and HPRT1- Rev2 primer for *HPRT1* expression evaluation. In addition, we also performed the amplification of the six different amplicons for *HPRT1* mentioned below by qPCR to compare the results with PCR Endpoint assays.

All experiments were carried out using the SensiFAST SYBR No-ROX Kit (Bioline) in a final volume of 10 µl containing: 5 µl of 2X SensiFAST SYBR No-ROX Mix, 0.4 µl of primer mix (400 nM final concentration), 4 µl of diluted cDNA and 0,6 µl of PCR-grade water. All PCRs were performed in triplicate from one sample taken from each preservation condition in all 20 patients included. PCR reactions were carried out in 96 well plates and using the CFX96 Real-Time PCR Detection System (Bio-Rad) following the steps: 95 °C for 2 minutes, 40 cycles of amplification (denaturation at 95 °C for 5 seconds, annealing for 10 seconds, and extension at 72 °C for 20 seconds) and final melt curve analysis from 65 °C to 95 °C to exclude unspecific PCR products.

The reproducibility and dynamic ranges for qPCR assays were determined by constructing a standard curve, beginning with 100 ng of RNA template as initial concentration and then serial diluted 1/5 until five linear concentration points. For Cq values acquisition, RFU threshold value was adjusted to 50 RFUs for each run.

### Statistics

All data generated from integrity and functional studies of RNA samples were analysed using multiple comparison tests: ANOVA test with Bonferroni correction to detect significant differences between procedures with at least a signification of *p* value <0.01. The spearman r index between RNA integrity Number (RIN) values derived from 2100 Bioanalyzer runs and Cq values were also calculated.

## Supplementary information

Supplementary Information.

Supplementary Information2.
