# Supplementary material for: Impact of different stabilization methods on RT-qPCR results using human lung tissue samples
Source: Sci Rep. 2020 Feb 27;10:3579. doi: 10.1038/s41598-020-60618-x (PMC7046779; doi:10.1038/s41598-020-60618-x)

SUPPLEMENTARY MATERIAL

Manuscript Title: Impact of different stabilization methods on RT-qPCR results using human lung tissue samples.

Authors

Margalida Esteva-Socias[1,2,3], Fernando Gómez-Romano [1,2,3], José Antonio Carrillo-Ávila[3,4], Alicia Loreto Sánchez-Navarro[1,2,3], Cristina Villena[1,2,3]*

1. Centro de Investigación Biomédica en Red in Respiratory Diseases (CIBERES), Plataforma Biobanco Pulmonar CIBERES, Hospital Universitari Son Espases, Palma, Spain.
2. Grupo de Inflamación, reparación y cáncer en enfermedades respiratorias, Institut d'Investigació Sanitària de les Illes Balears (IdISBa), Hospital Universitari Son Espases, Palma, Spain.
3. Spanish Biobank Network, Instituto de Salud Carlos III, Madrid, Spain
4. Andalusian Public Health System Biobank, Coordinating Node, Granada. Instituto de Investigación Biosanitaria ibs. Granada. Complejo Universitario de Granada/Universidad de Granada, Granada, Spain.

***Corresponding autor**

Cristina Villena Portella, PhD.

Table S1. Primers sequences, amplicon length and annealing temperatures.

Primer ID	Sequence	Amplicon length (bp)	Annealing temperature (ºC)
**HPRT1 - Fw**	CTGAGGATTTGGAAAGGGTGT	-	-
**HPRT1-Rev 1**	ACATCTCGAGCAAGACGTTC	73	61,8
**HPRT1-Rev 2**	AATCCAGCAGGTCAGCAAAG	156	63,6
**HPRT1-Rev 3**	TATGTCCCCTGTTGACTGGT	257	63
**HPRT1-Rev 4**	CTGCATTGTTTTGCCAGTGT	347	63
**HPRT1-Rev 5**	CTTGCGACCTTGACCATCTT	400	63,6
**HPRT1-Rev 6**	TATCCAACACTTCGTGGGGT	436	63,6
**SNRPD3 - Fw**	CCAGAGCCGAACTCTCTTCCT	-	-
**SNRPD3 - Rev**	GGTGCGTTCTTCAGCATGTC	269	65
**JUN - Fw**	GCCAGGTCGGCAGTATAGTC	-	-
**JUN - Rev**	GGACTCTGCCACTTGTCTCC	279	65

Figure S1. Full-length gels showing the HPRT1 PCR products for the amplicons tested in all preservation methods compared. At first stage of the study, a total of 7 amplicons of increasing length (73, 156, 257, 347, 400, 436 and 570 bp) were amplified, but the last one was not included in the final results. Thus, it is present in the uncropped gel images. Note that the results of the seventh amplicon were in concordance and do not interfere with the conclusions of the present study. M (molecular weight marker).

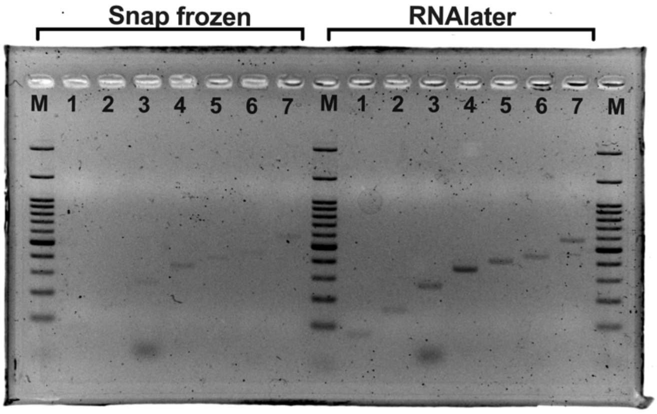

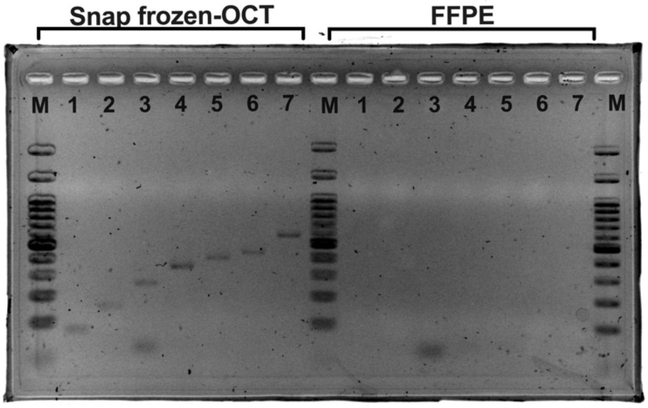

Figure S2. Amplification curves of *HPRT1*, *SNRPD3* and *Jun* genes for the 4 stabilization methods tested. RNL (salmon), SF (green), SF-OCT (blue) and FFPE (orange).

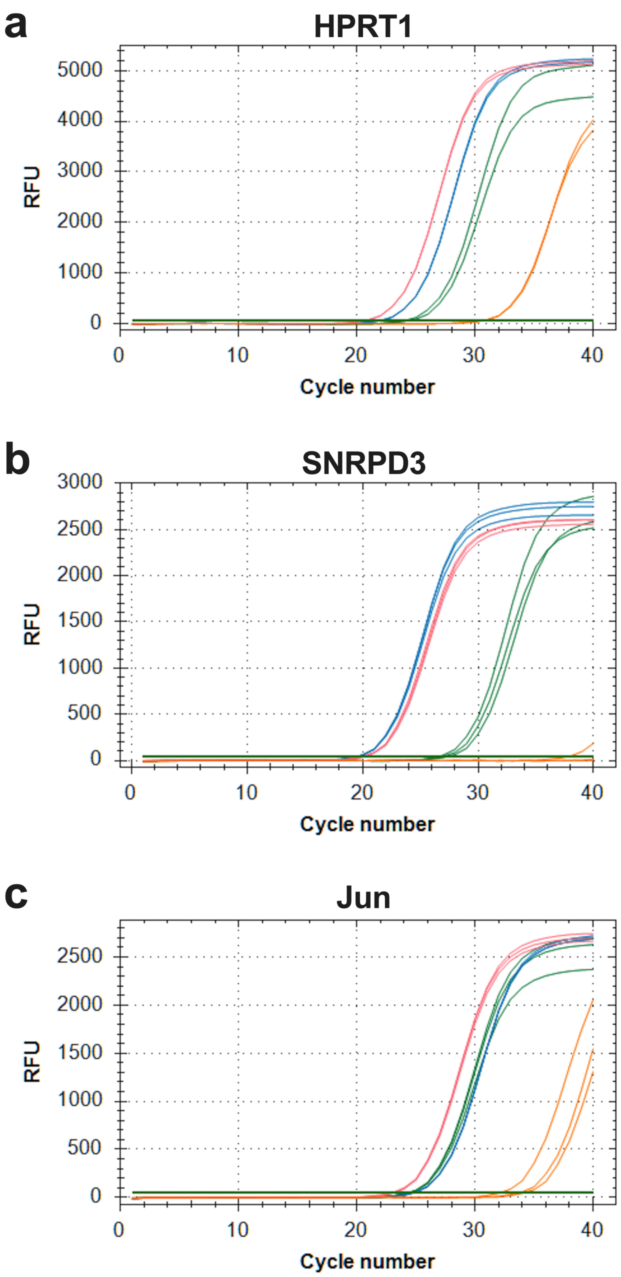

Figure S3. Fold change values calculated by Bioanalyzer 2100 during thawing time course. RNA derived from RNL tissue showed a stable behaviour among time, whereas RNA from SF and SF-OCT presented a noteworthy decrease in RIN number.

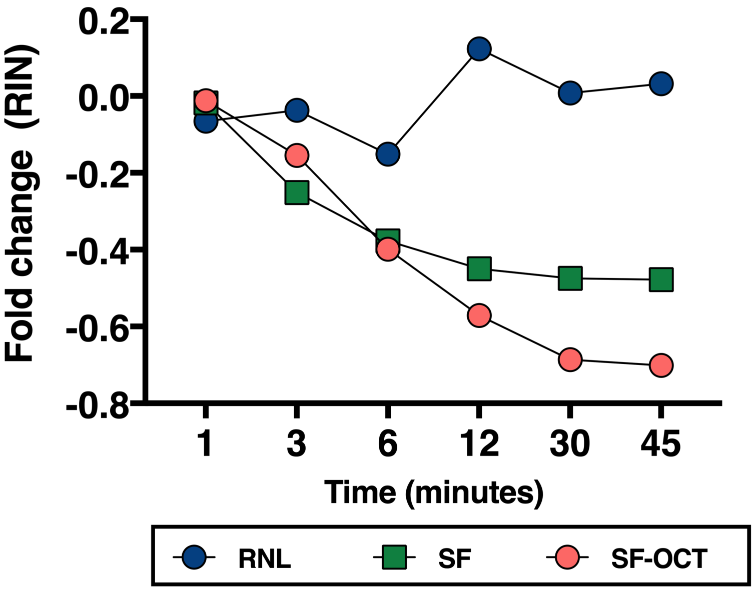

Figure S4. Comparative analysis between organ donation and surgical excision stratified by different preservation methods. No significant differences were found between organ donors (OD) and samples coming from patients undergoing to surgical excision (SE) regarding to *Jun* expression level. When RIN values were compared between types of collection, no differences were found, except in SF samples which showed statistically significant better quality in SE lung samples comparing with OD lungs ($p<0.001$).

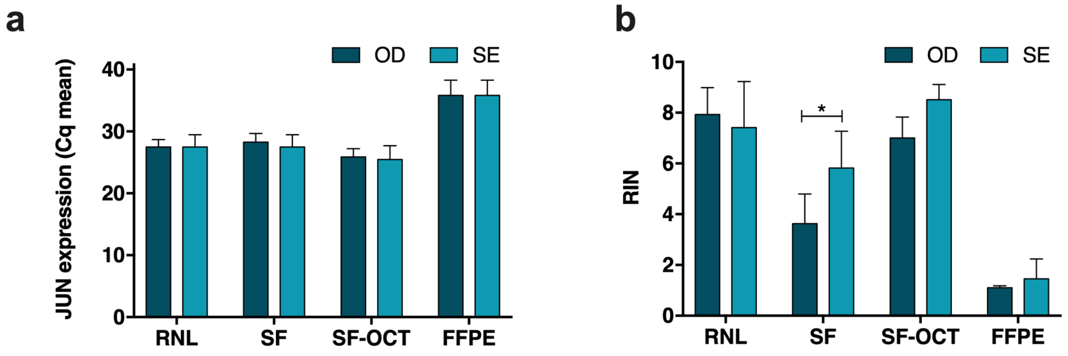

Supplement: Supplementary file 2 — Supplementary Information2. [file 41598_2020_60618_MOESM2_ESM.pdf]
